# Computational studies of a series of 2-substituted phenyl-2-oxo-, 2-hydroxyl- and 2-acylloxyethylsulfonamides as potent anti-fungal agents

**DOI:** 10.1016/j.heliyon.2020.e03724

**Published:** 2020-04-14

**Authors:** Yusuf Isyaku, Adamu Uzairu, Sani Uba

**Affiliations:** Department of Chemistry Ahmadu Bello University, P.M.B. 1044, Zaria, Nigeria

**Keywords:** Organic chemistry, Pharmaceutical chemistry, Theoretical chemistry, Ligand-based design, ADME/T, Molecular docking, Anti-fungal, Botrytis cinerea

## Abstract

Botrytis Cinerea is a plant pathogen that affect a large number of plant species like tomatoes, Lettuce, Grapes, and Strawberries among others. Sulfonamides are widely used in pharmaceutical industries as anti-cancer, anti-inflammatory and anti-viral agents. To complement our previous QSAR study, a ligand-based design and ADME/T study were carried out on these sulfonamides compounds for their fungicidal activity toward “Botrytis Cinerea”. With the help of AutoDock Vina version 4.0 in Pyrex software, the docking analysis was performed after optimization of the compounds at DFT/B3LYP/6-31G∗ quantum mechanical method using Spartan 14 softwar. Using the model generated in the previous QSAR work, the descriptors of the chosen model were considered in modifying the most promising compound ‘9’ in which twelve (12) derivatives were designed and found to have better activity than the template (compound 9). With compound 9j having the highest activity that turns out to be about 14 and 15 times more potent than the commercial fungicides “procymidone and chlorothalonil”. Furthermore, ADME/T properties of the designed compounds were calculated using the SwissADME online tool in which all the compounds were found to have good pharmacokinetic profile. Moreover, a molecular docking study on selected compounds of the dataset (compound 8, 13, 14, 19, 20, 21, 22 and 29) revealed that compound ‘20’ turned out to have the highest docking score of -8.5 kJ/mol. This compound has a strong affinity with the macromolecular target point (PDB ID: 3wh1) producing H-bond and hydrophobic interaction at the target point of amino acid residue. The molecular docking analysis gave an insight on the structure-based design of the new compounds with better activity against B. cinerea.

## 1 Introduction

Botrytis *cinerea* (gray mold), affects more than 200 dicotyledonous plant species and few other monocotyledonous plants found in moderate and subtropical regions (Williamson et al., 2007). It causes serious economic losses to both field and greenhouse-grown crops. The annual loss caused by B. cinerea earmarked to $10billion to $100billion per annual (Boddy, 2016). The symptoms of the disease varies across the organs and tissues of the plants. B. cinerea succeed in soft-rotten the fruit and leaves of the affected plant. Development of Brown lesions on undeveloped fruit and death of Twings are some of the signs of B. *cinerea* (NC State, 2018). The symptoms of the diseases are seeing at wound sites of the infected plants where the fungus begins to rot the plant. The gray mold on grapes may cause some respiratory allergic reaction called "winegrower's lung".

The fungus developed a series of strains to many commercial fungicides, thus, the need for developing novel antifungal agents with better activity and a novel mode of action to remedy the resistance of this fungus instead of commercial antifungal compounds. Also so, to have environmental friendly fungicides.

Sulfonamides exhibit a wide broad of activity in the pharmaceutical and agrochemical industries. Sulphur containing compounds like sulfonamides and dendrimers are widely used in medicine as anticancer, anti-viral, anti-bacterial, antiplasmodial and anti-inflammatory, among others (Kim et al., 2018; Fisher et al., 2018).

Due to an exclusive and series of strains developed by B. *cinerea* and a broad spectrum of sulfonyl compounds in anti-bacterial activities, there is a need for discovery of novel anti-B. *cinerea* compounds with better activity, in which here we brought a Computer-Aided Design of novel anti-B. *cinerea* with very high activity through template/ligand-based design in complement to our previous QSAR work (Isyaku et al., 2019) and also proposed a structure-based design of the same compounds using molecular docking analysis.

Ligand-based drug design also called indirect drug design (antifungal in this case) is an approach employed in the absence of 3D information of the receptor and it depends on the information of compounds that bind to the target enzymes of interest (Aparoy et al., 2012). Molecular docking help to investigate the capacity of the prepared compounds toward interaction with the protein residue of the target organism and to also predict the preferred orientation of the molecules.

This research aims to design series of novel compounds with better activity against B. *cinerea,* evaluate the designed compounds for their ADME/T properties and perform a molecular docking activity to explore the active site and to study the receptor-ligand interaction that will give us an insight toward the structure-based design of novel compounds against *Boytrytis Cinerea*.

## 2 Material and method

### 2.1 QSAR method of computer-aided drug design (CADD) in plant pathogen

#### 2.1.1 Optimization

From our previous work, compound 9 was chosen as the lead compound, modifications were made on it. Following the same procedure, where the 2D structures of the twelve (12) designed molecule were drawn using Chemdraw Ultra version 12.0 software and later transported to Spartan 14 software where they were converted to 3D structures before being optimized at Density Functional Theory/Becke, 3-parameter, Lee-Yang-Parr, and 6-31G∗ basis set (DFT/B3LYP/6-31G∗) and then converted to SDF format (Ibrahim et al., 2018; Abdullahi et al., 2018). The energies of the drawn molecules were minimized using Molecular Mechanics Force Field (MMFF) calculation to obtain low energy conformers (Arthur, 2016).

#### 2.1.2 Molecular descriptors calculations

Molecular descriptors are the properties of the molecule in numerical/mathematical values. PaDEL descriptor software was used to further calculate additional energy of those low energy conformers. Where a total of 1875 descriptors were calculated.

#### 2.1.3 The predicted activity of the designed compounds

Using the regression equation obtained from the best model of our previous work, the values of the model's descriptors were substituted in the equation to obtain the predicted activity of each of the designed compounds.

### 2.2 Theoretical prediction of ADME/T parameters

**ADMET Predictor** is a designed program of the computer for estimating pharmacokinetic parameters/properties of drug-like compounds from their molecular structures called the ADMET which referred to Absorption, Distribution, Metabolism, Excretion/Elimination, and Toxicity (Singh et al., 2013).

Being highly bioactive and low toxic by a drug/drug-like compound are not good enough criteria to qualify the compound as a good candidate. A better profile of pharmacokinetic is exclusively important for a novel compound that should be examined in the process of drug/drug-like compounds discovery. Hence, it is very significant to evaluate the ADMET profile of new compounds earlier to avoid waste of time/resources. Hence, we predicted the ADMET properties of our designed compounds (9a - 9l) using swissADME online software (Daina et al., 2017).

Lipinski et al. (1997) proposed four ADMET properties called the “Rule of Five”. This rule of five was the authentic and “most well-known rule-based filter” of drug-likeness which is used to examine whether a compound is well absorbed orally or not. The rule of five includes;

➢ Molecular weight (MW) ≤ 500
➢ Octanol/water partition coefficient (iLOGP **=** A log P) ≤ 5
➢ Number of hydrogen bond donors (HBDs) ≤ 5, and
➢ Number of hydrogen bond acceptors (HBAs) ≤ 10.6.

Under the Rule of Five, a molecule can only be orally active/absorb if it does not violate any two or more of the above rules. However, some complicated natural products are not suited to the rules. For that, many other drug-likeness rules/filters that equally as “Rule of Five” were proposed (Ghose et al., 1999; Bhal et al., 2007).

Hopkins in 2012 developed the QED (quantitative estimate of drug-likeness) concept (Bickerton et al., 2012) which generated eight physicochemical properties, which includes the four rules of five (MW, iLOGP, HBAs and HBDs) and four other parameters such as molecular polar surface area (TPSA), number of rotatable bonds (ROTBs), number of aromatic rings (nAROMs), and number of alerts for undesirable substructures (ALERTs i.e. PAINS #alert and Brenk #alert) using 771 marketed oral drugs (Bickerton et al., 2012). The concept of QED is the most flexible and adopted rules than ordinary drug-likeness rules. Table 1 represented some of the ADMET properties/parameters and their acceptable ranges.

**Table 1:** Some of the ADMET properties/parameters and their acceptable ranges.

Property name	Notation	Default range
MW	Molecular weight	50–500
iLOGP	octanol/water partition coefficient	-2–10
TPSA	Topological Polar Surface Area	20–130
HBA	Number of H-Bond acceptors	0–10
HBD	Number of H-bond Donors	0–5
RB	Rotatable bonds	0–5
nheavy atoms	Number of heavy atoms	15–50
logP	Lipophilicity of the compound	-0.7–5.0
MR	Molar refractivity	40–130

To evaluate the pharmacokinetic properties of the designed compounds, the 2D structure of the compounds were drawn on Chemdraw Ultra 12.0. Each structure was imported and the structure smiley was entered at the interface of the website (http://swissadme.ch/). The SwissADME drug design study was run and the ADMET properties/parameters were generated (Mishra, and Dahima, 2019).

### 2.3 Molecular docking studies

**Figure 1:**
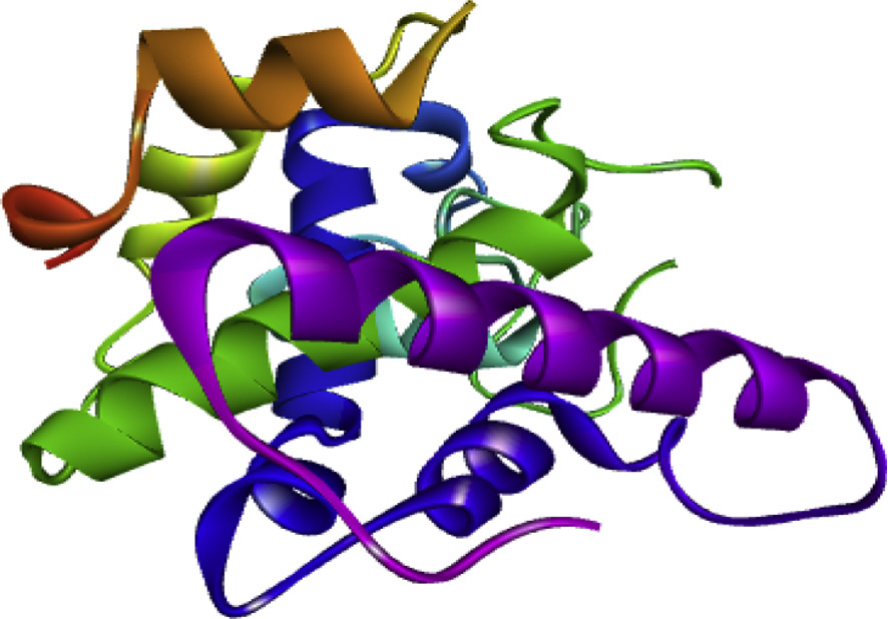
Prepared 3D structure of the receptor (3wh1).

**Figure 2:**
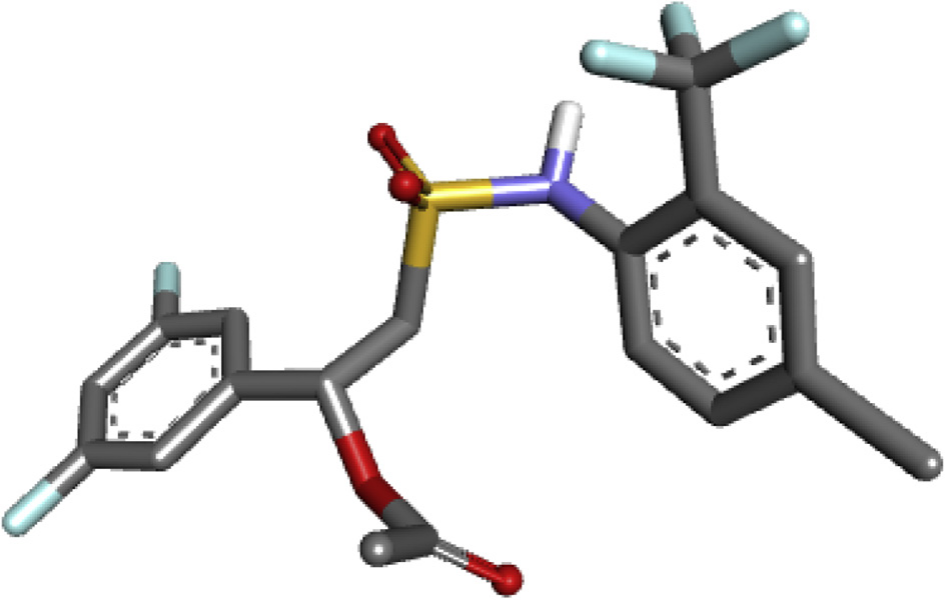
Prepared structure of the ligand.

**Figure 3:**
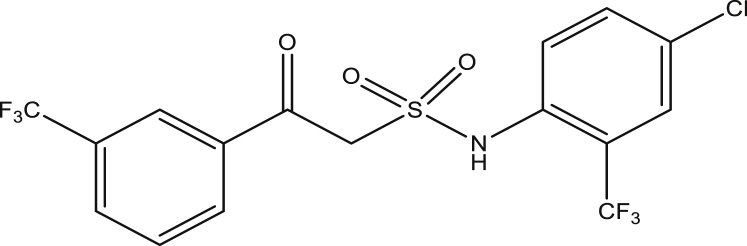
Chosen scaffold [compound 9 (pEC_50_ =0.858537)].

With aid of Autodock Vina of Pyrex software and the Discovery Studio, molecular docking study was performed between sulfonamide derivatives and the active site of *Bryum coronatum* (which has the similar active site with B. *cinerea*) (protein A9ZSX9, PDB ID: 3wh1) to examine an interaction between the binding pocket of the enzyme and the compounds (i.e. the ligands). A highly resolute crystal structure of *Bryum coronatum* was downloaded successfully from the protein databank (PDB ID: 3wh1). The downloaded substrate was carefully prepared using Discovery Studio which was later transported to the Pyrex for the docking calculation. With the aid of Spartan14 version 1.1.4, the optimized compounds (the ligands) were converted to PDB files (Parvatham et al., 2015). Subsequently, the prepared receptors alongside the prepared ligands were transported to Pyrex software for molecular docking study (Parvatham et al., 2015). The prepared receptors and prepared ligands were docked using Autodock Vina 4.2 integrated into the Pyrex software (Trott and Olson, 2010). Discovery Studio Visualizer was then used to visualize the docking results. The prepared receptor and prepared ligand are shown in Figures 1 and 2 (see Figure 3).

## 3 Results and discussion

### 3.1 Ligand-based design

The descriptors and mean effect of the best chosen QSAR model in our previous work were taken into consideration. Compound 9 was found to be the promising/lead compound, being the most predictive compound by the model, found within the domain of applicability of the model and also passes the Lipinski's rule of five.

**Figure 4:**
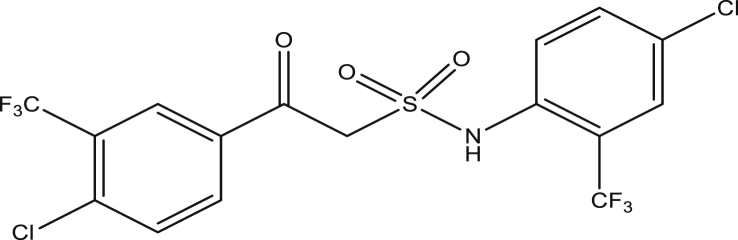
Compound 9a [N-(4-chloro-2-(trifluoromethyl)phenyl)-2-(4-chloro-3-(trifluoromethyl)phenyl)-2-oxoethanesulfonamide (pEC_50_0.6949857)].

**Figure 5:**
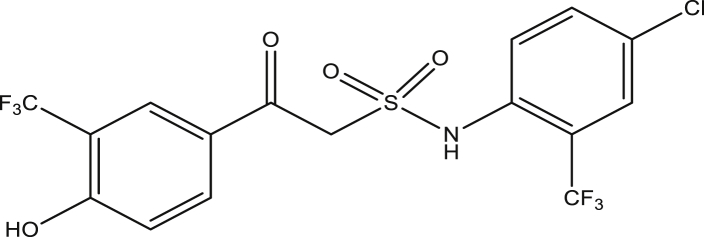
Compound 9b[N-(4-chloro-2-(trifluoromethyl)phenyl)-2-(4-hydroxy-3-(trifluoromethyl)phenyl)-2-oxoethanesulfonamide (pEC_50_0.1670010)].

**Figure 6:**
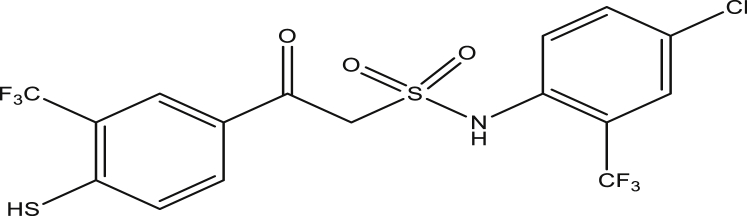
Compound 9c [N-(4-chloro-2-(trifluoromethyl)phenyl)-2-(4-mercapto-3-(trifluoromethyl)phenyl)-2-oxoethanesulfonamide(pEC_50_0.8472726)].

**Figure 7:**
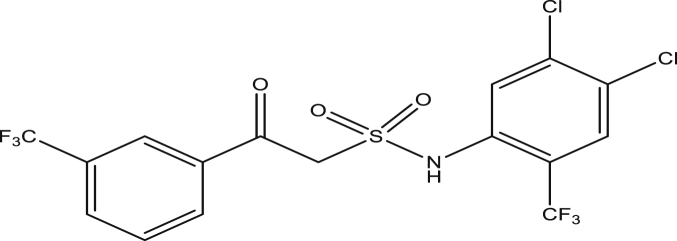
Compound 9d [N-(4,5-dichloro-2-(trifluoromethyl)phenyl)-2-oxo-2-(3-(trifluoromethyl)phenyl)ethanesulfonamide (pEC_50_0.4080003)].

**Figure 8:**
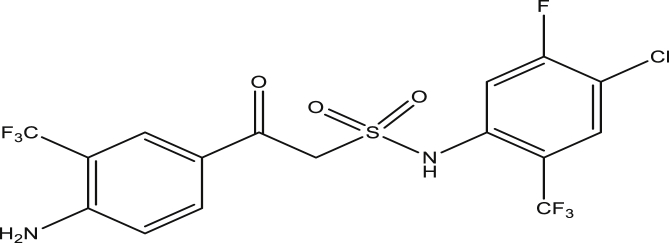
Compound 9e [2-(4-amino-3-(trifluoromethyl)phenyl)-N-(4-chloro-5-fluoro-2-(trifluoromethyl)phenyl)-2-oxoethanesulfonamide (pEC_50_0.7327058)].

**Figure 9:**
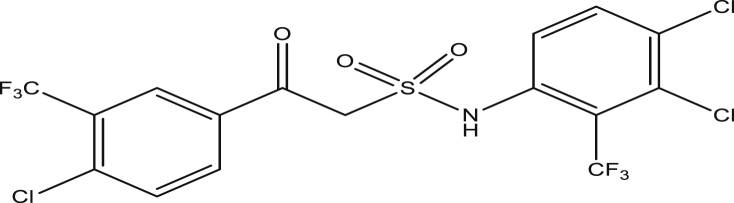
Compound 9f [2-(4-chloro-3-(trifluoromethyl)phenyl)-N-(3,4-dichloro-2-(trifluoromethyl)phenyl)-2-oxoethanesulfonamide (pEC_50_0.6040010)].

**Figure 10:**
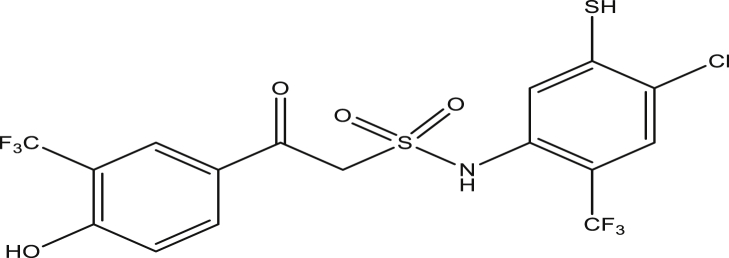
Compound 9g [N-(4-chloro-5-mercapto-2-(trifluoromethyl)phenyl)-2-(4-hydroxy-3-(trifluoromethyl)phenyl)-2-oxoethanesulfonamide (pEC_50_0.4591197)].

**Figure 11:**
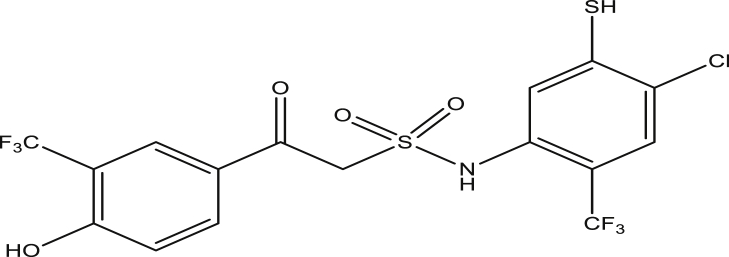
Compound 9h [N-(4-chloro-5-mercapto-2-(trifluoromethyl)phenyl)-2-(4-hydroxy-3-(trifluoromethyl)phenyl)-2-oxoethanesulfonamide (pEC_50_0.3187280)].

**Figure 12:**
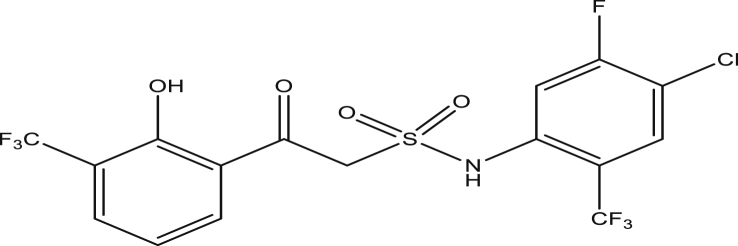
Compound 9i [N-(4-chloro-5-fluoro-2-(trifluoromethyl)phenyl)-2-(2-hydroxy-3-(trifluoromethyl)phenyl)-2-oxoethanesulfonamide (pEC_50_0.2065013)].

**Figure 13:**
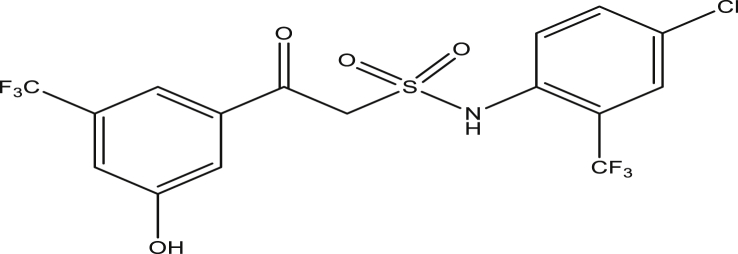
Compound 9j [N-(4-chloro-2-(trifluoromethyl)phenyl)-2-(3-hydroxy-5-(trifluoromethyl)phenyl)-2-oxoethanesulfonamide (pEC_50_0.071154)].

**Figure 14:**
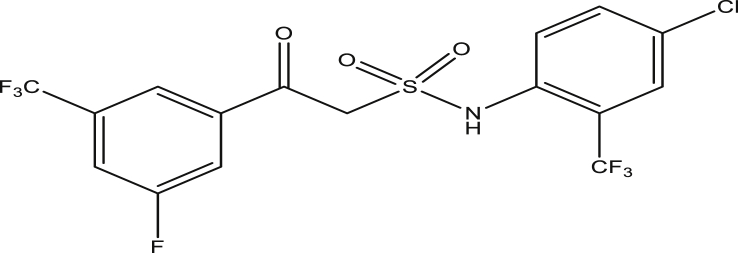
Compound 9k [N-(4-chloro-2-(trifluoromethyl)phenyl)-2-(3-fluoro-5-(trifluoromethyl)phenyl)-2-oxoethanesulfonamide (pEC_50_0.467899)].

**Figure 15:**
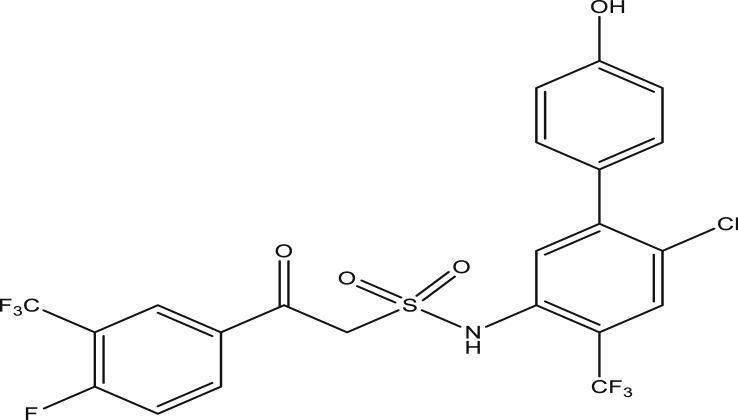
Compound 9l [N-(6-chloro-4′-hydroxy-4-(trifluoromethyl)-[1,1′-biphenyl]-3-yl)-2-(4-fluoro-3-(trifluoromethyl)phenyl)-2-oxoethanesulfonamide (pEC_50_0.396291)].

The modified compounds of compound 9 (designed compounds) were optimized and their molecular descriptors were calculated using paDEL descriptor software. For each of the designed compounds, these descriptors were then substituted in the regression equation of the selected model in which the values of the predictive activity of the compounds were calculated. All the designed compounds were found to be more potent with predictive activity values lower than the template (compound 9) which has pEC_50_ value of 0.858537 (7.22 in EC_50_). This low pEC_50_ value indicates low EC_50_ values which means more potent. From our previous work, we calculated the pEC_50_ as (pEC_50_ = -log1/EC_50_). With compounds 9a, 9c, 9e and 9f (Figures 4, 6, 8, and 9) being slightly high potent, having the pEC_50_ values of 0.694985703, 0.847272577, 0.732705894 and 0.604001056, while compound 9b, 9d, 9g, 9h, 9i, 9k and 9l (Figures 5, 7, 10, 11, 12, 14, and 15) are moderately higher. And compound 9j (Figure 13) recorded the highest potency with pEC_50_ value of 0.071154 (equivalent to 1.178023 in EC_50_) which is more than 6 times more active than the template (compound 9) and about 14 and 15 times more active than the commercial fungicides “procymidone and chlorothalonil” (with EC_50_ of 15.95 and 17.52 mg/L) as represented in Figures 4, 5, 6, 7, 8, 9, 10, 11, 12, 13, and 14 below.

### 3.2 Results of ADMET calculation

The most important and most difficult step in drug discovery and development (in which this account for the failure of about 60% of all drugs in the clinical phases.) is carrying out DMPK (drug metabolism and pharmacokinetics) studies, often referred to as ADMET (Mishra, and Dahima, 2019). In pharmacokinetic/pharmacology, ADMET stands for "absorption, distribution, metabolism, and excretion and toxicity”, in which they describes the disposition of a drug compound in the body.

ADMET Predictor is a designed program of a computer for estimating pharmacokinetic parameters/properties of drug-like compounds from their molecular structures called the ADMET (Singh et al., 2013).

Swiss ADME web tool is freely available software utilized to predict the physicochemical properties, absorption, distribution, metabolism, elimination and pharmacokinetic properties of molecules, which are key determinants for more clinical trials. It takes into account six physicochemical properties, which are very vital, like lipophilicity, flexibility, saturation, polarity, solubility, and size (Pires et al., 2015).

The result of the ADMET revealed physicochemical properties of the designed compounds which includes the rules of five (MW, iLOGP, HBAs and HBDs) and several other parameters/properties like molecular polar surface area (TPSA), number of rotatable bonds (ROTBs), number of aromatic heavy atoms, and number of alerts for undesirable substructures (i.e. PAINS #alert and Brenk #alert), among others as represented in the Table 2 below.

**Table 2:** Calculated ADME parameters of the designed compounds.

Comp	MW	iLOGP	HBD	HBA	TPSA	RB	nAH	MR	PAINS #alert	Brenk #alert
9a	480.21	2.35	1	9	71.62	7	12	94.45	0	0
9b	461.76	2.28	2	10	91.85	7	12	91.46	0	0
9c	477.83	2.25	1	9	110.42	7	12	96.69	0	1
9d	480.21	2.11	1	9	71.62	7	12	94.45	0	0
9e	460.78	1.86	2	9	97.64	7	12	93.85	0	1
9f	480.21	2.35	1	9	71.62	7	12	94.45	0	0
9g	512.27	1.94	1	9	110.42	7	12	101.7	0	1
9h	493.83	2.22	2	10	130.65	7	12	98.72	0	1
9i	479.75	1.8	2	11	91.85	7	12	91.42	0	0
9j	461.76	2.1	2	10	91.85	7	12	91.46	0	0
9k	463.73	2.61	1	10	71.62	7	12	89.4	0	0
9l	555.85	2.76	2	11	91.85	8	18	116.68	0	0

Molecular weight (MW), number of rotatable bonds (RB), number of hydrogen donors (HBD), number of hydrogen acceptors (HBA), Topological Polar Surface Area (TPSA), octanol/water partition coefficient (iLOGP), number of aromatic heavy atoms (nAH), Molar refractivity (MR) and the number of alerts for undesirable substructures/substructures (Brenk #alert and PAINS #alert) are presented in Table 2. According to Lipinski's rule of five and the concept of QED as presented in Table 1, all the designed compounds were in accordance with the rules by causing no more than one violation. That is to say, all the MW, RB, HBD, HBA, TPSA, iLOGP, nAH and MR are within the acceptable range. Also, there is no alert for PAINS and only 1 Brenk for compounds 9c, 9e, 9g and 9h, which indicated that the compounds are quite specific. Hence, we can now say that these designed and most active antifungal compounds (9a to 9l) possess a good pharmacokinetic profile.

### 3.3 Molecular docking study

A molecular docking study was performed between the compounds (those with the highest and moderate activity) and the crystal structure of *Bryum coronatum* (protein A9ZSX9, PDB ID: 3wh1). Molecular docking reveals two vital information: first is the correct conformation of a ligand-receptor complex and secondly, the binding affinity which represents an approximation of the binding free energy. Using the discovery studio, the interaction between the ligand and the binding pocket of the receptor such as aromatic, charge, H-bond and hydrophobic surface of the receptor were carefully studied and the pharmacophore of the molecule was identified and gave us an insight of what would enhance the inhibition activity of the designed compounds.

**Figure 16:**
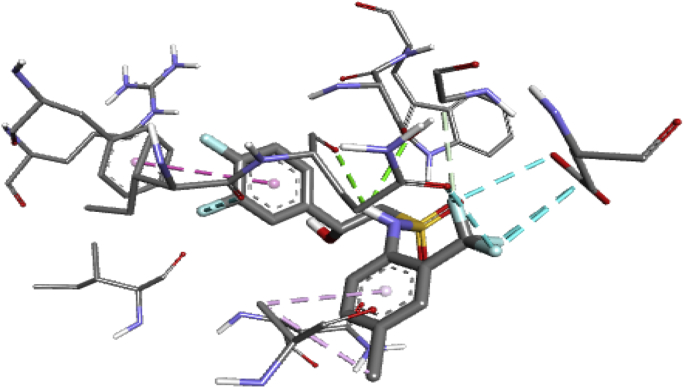
3D interaction between the compound with the highest docking score and receptor.

**Figure 17:**
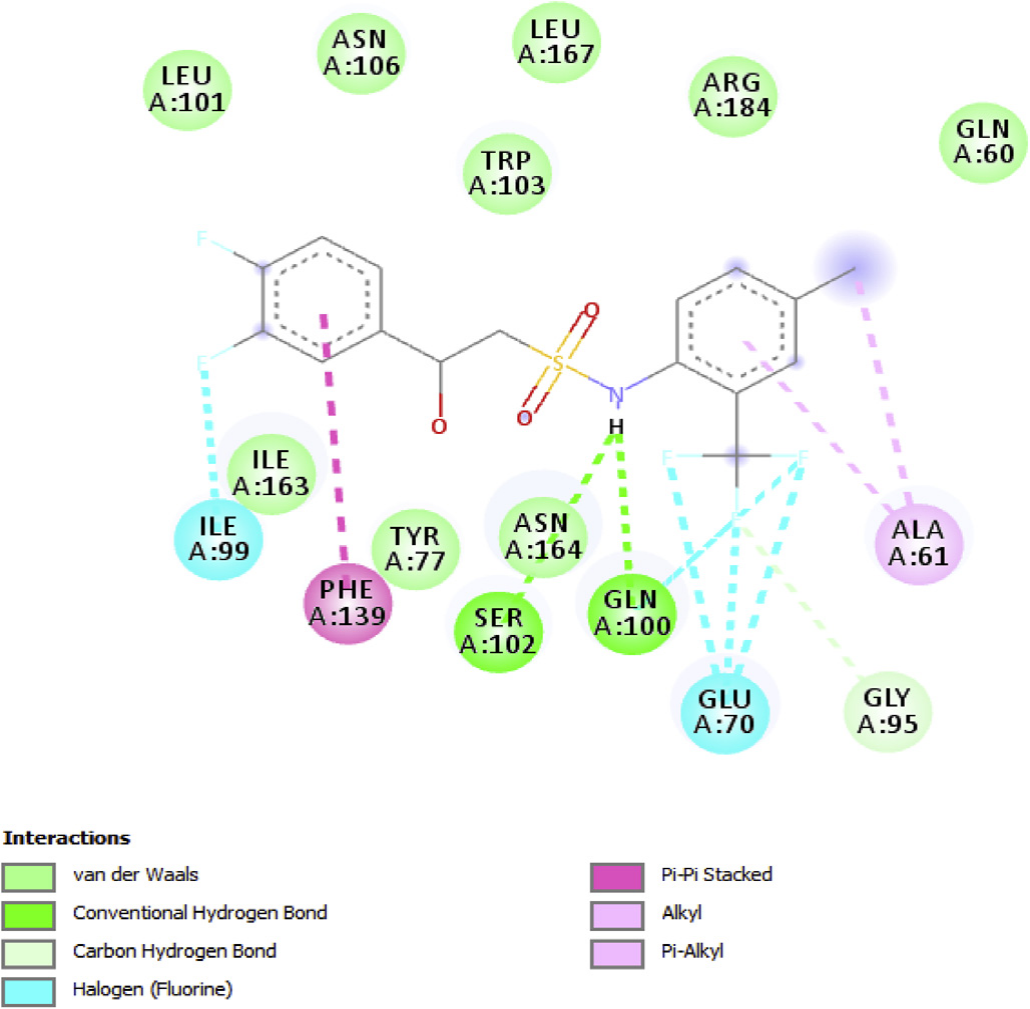
2D interaction of compound 20.

All the ligands show an interaction with the active site of fungus, that's to say they inhibit the activity of the fungus. Some ligands show high binding energy that varies from -7.3 to -8.5 kcalmol-1 as presented in Table 3. However, compounds 20 shows the highest binding score of -8.5 kcal/mol which is far more potent than the commercial fungicide “chlorothalonil” which had docking score of -6.1 kcal/mol. This compound (20) possessed SER102, ASN106, ARG184, GLN100, and ASN164 H-bond interaction with a bond length of 1.88667, 2.25994, 2.17112, 3.01763 and 1.99096 and hydrophobic interaction of PHE139, ALA61, ALA61, and ILE99. The interaction between the compound with the highest docking score and the binding pocket of the receptor is shown in Figure 16 while Figure 17 is the 2D hydrogen bond interaction of compounds 20 with the receptor. Table 3 indicates the binding affinity, hydrogen bond and hydrophobic interaction of the high docking score compounds.

**Table 3:** Ligands, binding affinity, H-bond and hydrophobic interaction between high binding score compounds and receptor.

Serial No.	Binding Affinity (kj/mol)	H-bond	H-bond length(Å)	Hydrophobic
8	-7.9	ARG184,ARG184, ASN164	2.57507,2.38318 2.45404	PHE139,ILE99, LEU101
13	-7.7	TRP103,ARG184	2.45454,2.92843	PHE139,GLN100, LEU101,ILE99, LEU101
14	-7.9	TRP103,ARG184	2.48547,2.4859	PHE139,ILE99, LEU101
19	-7.7	GLN60,TRP103, GLN180,GLU70	1.98605,2.58846, 2.66202,2.91443	PHE139,ILE99, ILE163
20	-8.5	SER102,ASN106, ARG184,GLN100, ASN164	1.88667,2.25994, 2.17112,3.01763, 1.99096	PHE139,ALA61, ALA61,ILE99
21	-8.0	GLN60,ASN106, ARG184,ARG184, ALA61,SER102, TRP103	2.66766,2.48774, 2.50626,2.95531, 2.13549,3.4367, 3.39742	PHE139,ALA61
22	-8.4	SER102,ASN106, ASN106,GLY165, GLY166,ARG184, ILE163,TRP103	2.17222,2.46585, 2.24396,2.47314, 2.69741,2.94555, 2.33241,2.18921	PHE139,LEU167, LEU167
29	-8.4	SER102,ASN106, ASN106,GLY165, GLY166,ILE163, TRP103,ILE163	2.11772,2.45099, 2.28368,2.44586, 2.60183,2.44328, 3.51076,3.48038	PHE139,LEU167, LEU167
Chlorothalonil	-6.1	ASN106,ASN106 ARG184	2.74625,2.98094 2.1097	ILE163,LEU167 TYR77,TRP103

## 4 Conclusion

Computer-Aided Drug Design (CADD) provides an invaluable method in lead identification and optimization. In this study, potent anti-fungal compounds were designed by employing template/ligand-based design based on our previous QSAR studies. Twelve compounds were designed in which all the twelve compounds were more potent than the template, in which compound 9j was found to have the highest activity which in comparison turn out to be more than 8 times than the template and about 14 and 15 times more potent than the commercial fungicides “procymidone and chlorothalonil”. Moreover, an ADME/T study on the designed compounds showed a good pharmacokinetics profile. Furthermore, a molecular docking study was carried out on the same compounds to give an insight into structure-based design. According to the docking scores, most of the ligands (compounds) show good inhibitory activity against the target enzyme where ligands 20 showed the highest binding affinity of -8.5 kcal/mol. This compound has a strong affinity with the macromolecular target point of the enzyme-producing H-bond and as well the hydrophobic interaction at the target point of amino acid residue.

## Declarations

### Author contribution statement

Yusuf Isyaku: Conceived and designed the experiments; Performed the experiments; Wrote the paper.

Adamu Uzairu: Analyzed and interpreted the data.

Sani Uba: Contributed reagents, materials, analysis tools or data.

### Funding statement

This research did not receive any specific grant from funding agencies in the public, commercial, or not-for-profit sectors.

### Competing interest statement

The authors declare no conflict of interest.

### Additional information

No additional information is available for this paper.
